# Treatment success in pragmatic randomised controlled trials: a review of trials funded by the UK Health Technology Assessment programme

**DOI:** 10.1186/1745-6215-12-109

**Published:** 2011-05-04

**Authors:** Louise Dent, James Raftery

**Affiliations:** 1 University of Southampton Clinical Trials Unit, MP131, Southampton General Hospital, SO16 6YD, UK; 2 NIHR Evaluation, Trials and Studies Coordinating Centre, University of Southampton, Southampton, SO16 7NS, UK

## Abstract

**Background:**

Previous research reviewed treatment success and whether the collective uncertainty principle is met in RCTs in the US National Cancer Institute portfolio. This paper classifies clinical trials funded by the UK HTA programme by results using the method applied to the US Cancer Institute trials, and compares the two portfolios.

**Methods:**

Data on all completed randomised controlled trials funded by the HTA programme 1993-2008 were extracted. Each trial's primary results was classified into six categories; 1) statistically significant in favour of the new treatment, 2) statistically significant in favour of the control treatment 3) true negative, 4) truly inconclusive, 5) inconclusive in favour of new treatment or 6) inconclusive in favour of control treatment. Trials were classified by comparing the 95% confidence interval for the difference in primary outcome to the difference specified in the sample size calculation. The results were compared with Djulbegovic's analysis of NCI trials.

**Results:**

Data from 51 superiority trials were included, involving over 48,000 participants and a range of diseases and interventions. 85 primary comparisons were available because some trials had more than two randomised arms or had several primary outcomes. The new treatment had superior results (whether significant or not) in 61% of the comparisons (52/85 95% CI 49.9% to 71.6%). The results were conclusive in 46% of the comparisons (19% statistically significant in favour of the new treatment, 5% statistically significant in favour of the control and 22% true negative). The results were classified as truly inconclusive (i.e. failed to answer the question asked) for 24% of comparisons (20/85). HTA trials included fewer truly inconclusive and statistically significant results and more results rated as true negative than NCI trials.

**Conclusions:**

The pattern of results in HTA trials is similar to that of the National Cancer Institute portfolio. Differences that existed were plausible given the differences in the types of trials -HTA trials are more pragmatic. The results indicate HTA trials are compatible with equipoise. This classification usefully summarises the results from clinical trials and enables comparisons of different portfolios of trials.

## Background

Ethically an RCT should only be undertaken if clinicians are truly unsure which of the interventions being compared is more likely to benefit patients [1,2]. This concept is referred to as equipoise or the collective uncertainty principle [1,3]. The "equipoise hypothesis" implies a predictable relationship between equipoise and the ultimate outcomes of trials [4,5]. Given a random unbiased sample of trials, no significant difference would be expected in the proportion favouring the new treatment to the proportion favouring the standard treatment [3,6,7].

The pattern of results and satisfaction of equipoise in a cohort of RCTs from the US National Cancer Institute portfolio [3,8-10] was reviewed by Djulbegovic et al covering 743 National Cancer Institute trials conducted 1955-2000. They classified the primary outcome trial results into one of six categories based on whether they included an important difference in favour of the new or standard treatment [8] 24% of trial results had statistically significant results in favour of the new treatment and the new treatment was favoured by the researchers in 41% of comparisons. They concluded that about 25% to 50% of new cancer treatments assessed in RCTs will be successful. Soares, Kumar and Joffe conducted similar reviews on NCI trials with similar results [3,9,10]. Djulbegovic et al were the only authors to apply the six category classification of primary outcome results.

Johnson et al found most people would accept an RCT to be ethical if the probability of success of a new treatment is between 50% and 70% [11]. Djulbegovic et al found most members of an institutional review board would approve a trial with expected probability of success of a new treatment between 40% and 60% [12].

The Health Technology Assessment programme (HTA) of the National Institute for Health Research is the leading public funder of randomised controlled trials (RCTs) in the NHS [13,14]. It funded and published the results of 74 trials between 1993- 2008. These trials aim to answer questions of importance to the NHS, usually evaluate cost as well as clinical effectiveness and inform NICE decisions. The trials involve a wide range of interventions, areas of health and usually include patient reported outcomes.

This paper classifies the HTA trials by results using the classification developed by Djulbegovic et al [8].

## Aims

1. To classify HTA superiority trials by results using Djulbegovic's classification

2. To compare the results with those from the similar classification of NCI trials

## Methods

### Trials included

All randomised controlled trials (RCT) funded by the HTA programme were eligible for inclusion in the study (13). The trial had to have had a superiority design and have published their results by May 2008. Trials were excluded if the primary outcome results were unclear or lacked a confidence interval. Comparisons were excluded if it was unclear which trial arm was the control.

### Data extracted

We extracted data on each trial from the HTA journal series publication on: trial design, trial interventions, no. of arms, primary outcome(s), primary time point, sample size calculation parameters including the minimum clinically important difference that the trial aimed to detect, sample size planned and achieved and primary outcome results from the primary time point including 95% confidence interval (CI) for the treatment difference (defined as the primary comparison).

We defined the primary outcome as:

"Primary outcome - Main outcome(s) of interest, in the following hierarchical order:

1. Explicitly defined as primary or main

2. Outcome used in the power calculation

3. Main outcome stated in the trial objectives"

Chan et al [15].

The primary timepoint was defined similarly:

"Primary time point - Main follow up timepoint(s) of interest, in the following hierarchical order:

1. Explicitly defined as primary or main follow up time point of interest

2. Time point specified in the power calculation

3. Main follow up timepoint stated in the trial objectives

If the authors didn't state a primary timepoint we defined it as the first follow up time point for the primary outcome. The first timepoint was selected because this is when the trial was expected to have greatest power (as smallest loss of participants). As this could bias our analysis in favour of more conclusive results we also conducted a sensitivity analysis selecting the last follow up time point for the primary outcome as "primary timepoint".

If the authors included a number of sample size calculations for the primary outcome (for example because they had re-estimated the required sample size part way through the trial), the smallest clinically meaningful difference specified in the calculations was extracted.

### Data extraction process

Data was extracted by a research assistant and 100% checked by LD. Discrepancies were resolved by discussion with JR.

### Classification of data

We classified each of the trial intervention arms as either new/experimental intervention or the control/standard intervention. An intervention was defined as the control intervention if it was described by the authors in the journal series as either "standard care, usual care, control, placebo or the intervention used most frequently in the NHS". A public health consultant independent of the project team, Andrew Cook, checked the classification.

**Figure 1:**
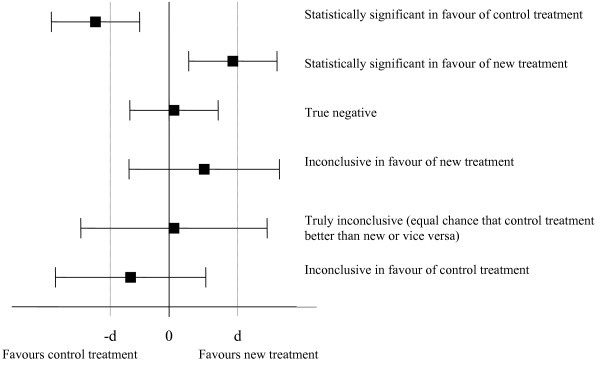
**Classification of RCT results based on 95% confidence interval and minimum important difference from the trial sample size calculation (d)**. 1. Explanation of the labels: The label given to each result are the labels used by Djulbegovic et al [8]. The labels assigned to the two inconclusive categories have been slightly amended from that used by Djulbeovic. 2. Explanation of -d and d: Djulbegovic's method used -d = 0.8 and d = 1.2 for all trials reviewed because all outcomes they evaluated were binary and expressed as a hazard ratio, odds ratio or relative risk. The value used for -d and d in this review was the minimum important difference specified in the sample size calculation from each trial. For example if the sample size calculation for a trial indicated an increase of 5 points was the minimum important difference for the primary outcome then -d was equal to -5 and d was equal to 5. 3. If the primary outcome result was expressed as a Hazard Ratio, Relative Risk or Odds Ratio then zero in this figure is replaced with 1

For each trial primary comparison, we classified the results into one of the six categories developed by Djulbegovic et al 1) statistically significant in favour of the new treatment, 2) statistically significant in favour of the control treatment 3) true negative, 4) truly inconclusive, 5) inconclusive in favour of new treatment or 6) inconclusive in favour of the control treatment [8]. We decided which category by comparing the 95% confidence interval for the difference in primary outcome to the difference specified in the sample size calculation. See figure 1 for an illustration of which category was assigned in each situation. The results were classified as true negative if the 95% confidence interval excluded a meaningful difference in either direction implying the treatments have similar effect (figure 1). The results were classified as truly inconclusive if the 95% confidence included a meaningful difference in either direction (i.e. trial failed to answer the primary question).

For example, one of the trials examined the effectiveness of short-term counselling in general practice for patients with chronic depression or combined depression and anxiety, compared with general practitioner (GP) care alone. The primary outcome of the trial was the Beck Depression Inventory (BDI) and the sample size calculation specified the minimum important difference in BDI was 3.5 with a lower BDI score implying better outcome. The primary results at 12 months found the mean difference in BDI score between counselling and GP care alone was 1.18 with a 95% confidence interval of -1.56 to 3.92. This result was classified as "Inconclusive in favour of control" the last row in figure 1. This is because "d" for this trial was -3.5 (the aim with the intervention was that it would reduce BDI score but it actually increased it on average very slightly and the confidence interval included an increase of 3.5 points).

We used the difference from the sample size calculation rather than the surrogate (global proxy) of 0.8 and 1.2 used by Djulbegovic for three reasons. First, the primary outcome in the HTA trials was rarely a hazard ratio, odds ratio or relative risk, unlike in the NCI trials. If the primary outcome results were presented as an odds ratio, hazard ratio or relative risk and a minimum important difference in relation to these wasn't discussed in the sample size calculation then we used 0.8 and 1.2 as a proxy for a clinically meaningful difference as per Djulbegovic et al.

Second, it can be argued that the difference specified by the original researchers in the sample size calculation represents a minimum important difference. This may not always be true, as investigators may specify a difference relating to the sample size they can achieve [16]. However, as the average effect size the trials aimed to detect was 0.29, considered small by Cohen (range 0.06 to 0.67, median 0.28, inter-quartile range 0.18 to 0.40), we believe this is not true with these trials.

Third, we considered that using the actual important difference specified is more reliable than converting all trial results to a common outcome and using a global proxy.

One of the trials resulted in 12 primary comparisons because it had 6 domains of SF36 as primary outcome and two active arms, both being compared to a control arm. To ensure this trial didn't dominate results we conducted a sensitivity analysis including 2 primary comparisons for this trial one for each comparison of active arm to control. We selected the two primary comparisons as those relating to the category which applied most frequently.

### Analysis of data

We summarised the characteristics of the trials and comparisons included. We calculated the proportion of trials which achieved the required sample size and 80% of the required sample size.

We calculated the proportion of comparisons which favoured the new treatment and the proportion which favoured the control treatment (regardless of whether the results were statistically significant). We calculated an exact binomial 95% CI for the proportion of treatments which favoured the new intervention [17]. If the 95% CI included 50% we concluded the results were consistent with equipoise [3].

We calculated the percentage of comparisons in each of the six categories and from this the percentage of comparisons which were conclusive. A comparison was defined as conclusive if it was either statistically significant or true negative (categories 1, 2 or 3 in Figure 1).

We compared the percentage of trial results in each category to the percentage in each category from the Djulbegovic analysis of NCI trials [8].

We conducted a sensitivity analysis including trial results that related to the longest follow up for trials which didn't specify a primary time point and including 2 results only for the trial which involved 12 primary comparisons.

## Results

### Included and excluded RCTs

**Figure 2:**
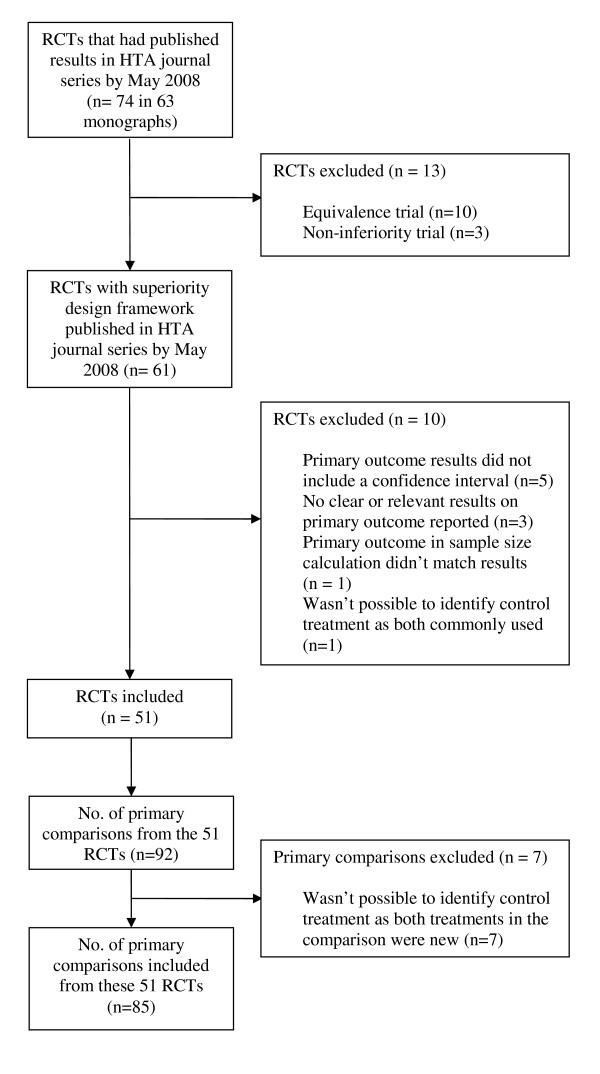
**Included and excluded RCTs and comparisons**.

Between 1993 and May 2008 the UK HTA programme funded and published the results from 74 RCTs in the HTA monograph series in which all its trials are reported [18]. Of the 74 trials, thirteen were excluded because they were equivalence or non-inferiority trials and a further ten because relevant data were not available (figure 2).

### Characteristics of included RCTs and comparisons

The 51 superiority trials which involved over 48,000 participants had 85 primary comparisons because some included more than two randomised arms or had several primary outcomes (additional file 1, appendix 1 shows the breakdown of comparisons to trials).

The characteristics of the trials and comparisons are shown in tables 1- 2 (and additional file 2, appendix 2). Most trials were parallel design (96%) and involved 2 arms (75%). The trials evaluated a wide range of interventions and diseases. Service delivery and surgery were the most frequent type of intervention evaluated (22% and 16% respectively). The disease investigated in 48% of the trials were diseases of the nervous system, circulatory system and musculoskeletal system and connective tissue. The most frequent type of primary outcome used in the trials was a symptom score or measurement of depression/pain. The median number of participants in each trial was 457 and only 67% of trials achieved 80% of their planned sample size. Most trials performed an intention to treat analysis (94%), but in 3 trials the analysis population used was unclear. Blinding was frequently not possible and most trials didn't blind participants (96%), the person administering the intervention (98%) or the outcome assessor (75%). Of the 13 which blinded the outcome assessor, a number reported this was not always successful because they could not stop the participant revealing the intervention to the assessor.

**Table 1:** Characteristics of the 51 included trials

Characteristic	No. of trials (%)
**Study design**	
Parallel	49 (96)
Factorial	2 (4)
**Number of arms**	
2 arms	38 (75)
3 arms	8 (16)
4 arms	2 (4)
5 arms	3 (6)
**No. of participants**	
Total number of participants in all trials	48,323
Mean number of participants per trial (excluding outlier involving 14,802 participants)	670
Median number of participants per trial (IQR)	457 (212 to 806)
**Trials achieving sample size**	
Trials achieving at least required sample size	18 (35%)
Trials recruiting 80% of original target	34 (67%)
**Intervention evaluated**	
Service Delivery	11 (22)
Surgery	8 (16)
Psychological Therapy	5 (10)
Physical Therapies	5 (10)
Diagnostic	5 (10)
Drug	4 (8)
Devices	4 (8)
Social Care	3 (6)
Education and Training	2 (4)
Complementary Therapies	2 (4)
Vaccines and Biologicals	1 (2)
Diet	1 (2)
**Disease (ICD10 chapter)**	
M00-M99 Diseases of the musculoskeletal system and connective tissue	8 (16)
G00-G99 Diseases of the nervous system	8 (16)
I00-I99 Diseases of the circulatory system	8 (16)
Z00-Z99 Factors influencing health status and contact with health services	6 (12)
F00-F99 Mental and behavioural disorders	6 (12)
O00-O99 Pregnancy, childbirth and the puerperium	4 (8)
K00-K93 Diseases of the digestive system	3 (6)
N00-N99 Diseases of the genitourinary system,	3 (6)
C00-D48 Neoplasms	2 (4)
LOO-L99 Diseases of the skin and subcutaneous tissue	1 (2)
P00-P96 Certain conditions originating in the perinatal Period	1 (2)
S00-T98 Injury, poisoning and certain other consequences of external causes	1 (2)
Primary outcome types in the 51 trials*	
Symptom score or measurement of depression/pain	13 (25)
Quality of life measure (including generic and disease specific)	10 (19)
Positive event rate (e.g. improvement in symptoms)	10 (19)
Adverse event rate (e.g. post operative nausea and vomiting)	9 (17)
Survival/mortality	6 (11)
Measurement of function	4 (8)
Other	1 (2)
**Intention to treat analysis**	
Yes	48 (94)
No	0 (0)
Unclear	3 (6)
**Blinding / Masking**	
Participant blinded	2 (4)
Outcome assessor blinded	13 (25)
Person administering intervention blinded	1 (2)
No blinding	38 (74)
**Combination of blinding / masking**	
Participant and outcome assessor blinded only	2 (4)
Administrator and outcome assessor blinded only	1 (2)
Outcome assessor blinded only	10 (20)
No blinding	38 (74)

*This adds up to 53 because two trials included 2 different types of primary outcome

**Table 2:** Characteristics of the 85 comparisons from the 51 included trials

Characteristic	No. of comparisons (%)
**Type of comparison**	
One active treatment vs. other active treatment	72 (85)
Active treatment vs. placebo/no treatment	13 (15)

### Trial results

Across all comparisons, the new treatment had superior results (whether significant or not) in 61% of the comparisons (52/85), with 95% exact confidence interval 49.9% to 71.6%. This confidence interval just includes 50% implying the results are compatible with equipoise (as per Kumar's hypothesis) [3]. The confidence interval is also compatible with Djulbegovic et al and Johnson et al's suggested range for an RCT to be ethical [11,12].

**Figure 3:**
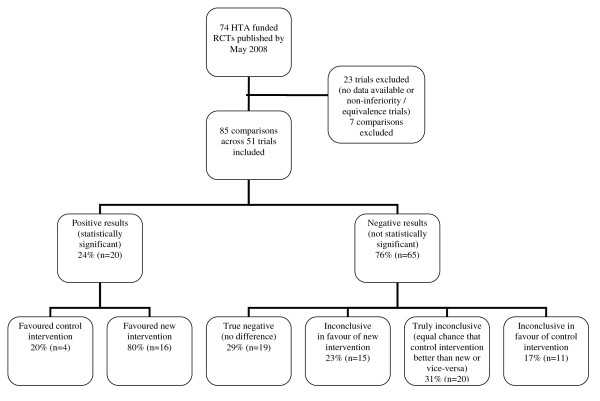
**Classification of primary outcome results of the HTA trials**.

Overall, 46% of comparisons were conclusive (figure 3 categories 1, 2 and 3). Twenty four percent of results were statistically significant (20/85), of which 80% favoured the new treatment and 20% the control. Twenty two percent were rated as true negative with the 95% confidence interval excluding the possibility of an important difference in either direction. The results were classified as truly inconclusive for 24% of comparisons (20/85). For these comparisons the 95% confidence interval still included the possibility of an important difference in either direction.

Of the 20 truly inconclusive comparisons, twelve were from trials which failed to recruit the planned sample size, 5 of which failed to recruit 80% of their target. A similar percentage failed to recruit across the other categories so failing to recruit cannot be concluded as the sole reason these trial results were truly inconclusive.

### Sensitivity analysis

**Figure 4:**
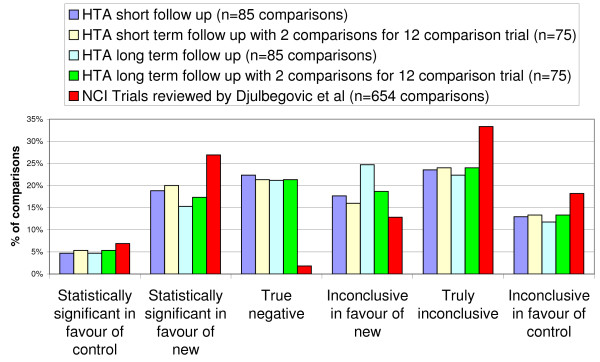
**Primary outcome results of the HTA trials compared to NCI trials with sensitivity analysis**.

The sensitivity analyses show that the percentage of comparisons included in each category does not change significantly depending on whether the shortest or longest follow up results were included or if the number of comparisons for one trial were reduced (figure 4). The results are therefore not sensitive to inclusion criteria.

### Comparison to NCI results

Comparing the percentage of results in each category to the review of NCI trials shows, HTA trials involved proportionally fewer trials with statistically significant results, fewer trials with truly inconclusive results and more trials with "True Negative" results (figure 4, table 3) (statistically significant comparisons - 34% NCI vs. 24% HTA, "Truly inconclusive" comparisons - 33% NCI (218/654) versus 24% HTA (20/85), "True negative" comparisons - 2% NCI (12/654) versus 22% HTA (19/85)).

**Table 3:** Table of results used to produce figure 4

	NCI Trials reviewed by Djulbegovic et al (n = 654 comparisons)		HTA short follow up (n = 85 comparisons)		HTA short follow up with 2 comparisons for 12 comparison trial (n = 77)		HTA long term follow up (n = 85 comparisons)		HTA long term follow up with 2 comparisons for 12 comparison trial (n = 75)	
Classification / category	N	%	n	%	n	%	n	%	n	%
1. Statistically significant in favour of control	45	7%	4	5%	4	5%	4	5%	4	5%
2. Statistically significant in favour of new	176	27%	16	19%	15	20%	13	15%	13	17%
3. True negative	12	2%	19	22%	16	21%	18	21%	16	21%
4. Inconclusive in favour of new	84	13%	15	18%	12	16%	21	25%	14	19%
5. Truly inconclusive	218	33%	20	24%	18	24%	19	22%	18	24%
6. Inconclusive in favour of control	119	18%	11	13%	10	13%	10	12%	10	13%
**Total**	654	100%	85	100%	75	100%	85	100%	75	100%

## Discussion

### What we found

In the HTA trials we found a similar proportion of results favouring the new treatment as in the analysis of the NCI trials. Approximately half of the primary results were conclusive and a quarter were truly inconclusive. HTA trials included fewer truly inconclusive and statistically significant results and more results rated as true negative than NCI trials.

### Strengths and weaknesses

This is the first study that we are aware of to assess treatment success and equipoise in a cohort of pragmatic RCTs. Publication bias did not apply as all HTA funded trials are published in the open access HTA monograph series regardless of study results.

Our study has limitations. Firstly, HTA trials often include multiple primary outcomes which are measured at multiple time points. Therefore, selecting which trial result was the primary was not always straight forward. However, having access to a comprehensive report of the trial meant we had access to all the data and trial results. Where there was uncertainty a sensitivity analysis was performed and showed that this made no difference to the main results.

Second our analysis kept the primary outcome results in the original unit of measurement and compared them to the difference specified by the original researchers in the sample size calculation. We assumed that the difference specified by the original researchers in the sample size calculation represents the minimum important difference for that outcome in that population. There is some evidence from other trials that this is not always the case, with investigators specifying the difference relating to the sample size they can achieve [16]. However, as the average effect size the trials aimed to detect was 0.29, considered small by Cohen (range 0.06 to 0.67, median 0.28, inter-quartile range 0.18 to 0.40), we believe this is not true with these trials. Further, we believe using the actual important difference specified is more reliable than converting all trial results to a common outcome and using a global proxy.

### Comparison with other studies

Our results were similar to previous reviews of oncology RCTs [8-10,16,19-21]. Where differences exist these seem to be justified given the differences in the types of trials funded by HTA and NCI. HTA trials are generally more pragmatic than NCI trials [22].

By comparison with the NCI trials in Djulbegovic et al, HTA trials included fewer truly inconclusive and statistically significant results and more results rated as true negative. Three reasons might explain these differences. Firstly, the HTA trials were on average almost twice as large as the trials included in the NCI cohort (mean number of participants 670 for HTA trials and 347 for NCI trials). A larger sample size results in narrower confidence intervals which could account for why HTA trials have more results graded as true negative instead of truly inconclusive when compared to NCI trials. Secondly, these narrow CI's are more likely to be centred around a smaller difference in HTA trials because their pragmatic nature conducted in a real life NHS setting dilutes any treatment effect. This could be why there are fewer statistically significant results and more true negative results in HTA trials than NCI trials. Thirdly, the method used to assign each trial result to one of the six categories was slightly different; Djulbegovic used a global proxy for an important difference whereas we used a trial specific measure where possible.

### Meaning of the study

The fact that only 24% of the HTA trial results were statistically significant is similar to other studies. Djulbegovic, Joffe, Kumar and Soares who reviewed National Cancer Institute trials found the percentage of statistically significant results was 34% (221/654), 32% (33/103), 29% (44/152), and 12% (7/52) respectively [3,8-10]. Of those the percentage which favoured the experimental treatment was 80% (176/221), 90% (30/33), 72% (32/44) and 86% (6/7) respectively. All of these studies concluded the results were consistent with equipoise.

Just over half of the HTA trials reviewed and three quarters of NCI trials had inconclusive results. A trial result could be inconclusive due to one or more of the following reasons:

1. If the trial didn't recruit the planned sample size

2. If the difference observed was smaller than difference the trial aimed to detect (as specified in the sample size calculation)

3. If the variability in primary outcome was greater than anticipated in the sample size calculation or the control group event rate was larger than anticipated in the sample size calculation (both of which would require a larger sample size than planned)

4. If a significant difference wasn't observed simply due to chance. Most trials are powered in the sample size calculation for 80% power which implies there is a 1 in 5 chance the trial wont find a statistically significant difference if it exists.

Djulbegovic stated that the reasons for such a large number of truly inconclusive NCI trials was not entirely clear, but did not appear to be due to accrual problems but due to the overly optimistic size of the treatment effect that they designed their trials to measure (hence the trials were not designed to be big enough to detect important differences) [8]. The reason for such a large number of trials having truly inconclusive results in HTA also does not appear to be solely due to accrual problems as a similar percentage of trials with conclusive results as inconclusive results failed to recruit to target. In trials which recruited the required sample size but still had inconclusive results the difference observed must either have been smaller than expected or variability greater (situations 2 to 4 above).

### Unanswered questions and further research

Further work might usefully review the conclusions of HTA trials with truly inconclusive results and undertake a literature search to assess whether another trial is needed to answer the question.

This study focused solely on the clinical primary outcome results and did not consider other outcomes or economic analysis results which are important in HTA trials. Previous research has assessed equipoise in a number of ways including converting all results to the same scale and applying meta-analysis and reviewing trial conclusions. Our study did not apply these methods because we were keen to keep the results in the original scale of measurement and not lose their meaning and because of the additional complexity of conclusions in relation to health economic analyses conducted in HTA trials. Further research in this area could usefully systematically review the authors conclusions about which intervention was preferred which would take into account multiple outcomes, benefits, harms and costs of each intervention. Further work might also systematically review the economic results of these trials and compare them with the clinical results.

The seemingly low success rate predicted by the equipoise hypothesis and observed in this and Djulbegovic [8], only applies to publicly funded trials. It might not hold in industry sponsored trials on the grounds that industry invest heavily in their drug development programs, have a better knowledge of which drugs work and as a result have a better success rate. This, however, has not been confirmed but is a testable hypothesis. We would encourage others, particularly industry, to conduct such a study.

## Conclusions

The results indicate HTA trials are compatible with equipoise (the new treatment had superior results in 61% of the comparisons 95% CI 49.9% to 71.6%). The pattern of results in HTA trials is similar to that of the National Cancer Institute portfolio. Differences that existed were plausible given the differences in the types of trials -HTA trials are more pragmatic.

The classification of trials by results developed by Djulbegovic is a useful way of summarising the results from clinical trials. Our application of it, allows the classification to be applied to trials with different outcomes. We look forward to comparisons with other groups of trials, particularly trials funded by industry.

## Abbreviations

NETSCC: NIHR Evaluation Trials and Studies Coordinating Centre (http://www.netscc.ac.uk); NIHR: NHS National Institute for Health Research (http://www.nihr.ac.uk); NIHR HTA: NHS National Institute for Health Research Health Technology Assessment programme (http://www.hta.ac.uk); RCT: Randomised controlled trial; UK: United Kingdom; USA: United States of America; NICE: National Institute for Health and Clinical Excellence (http://www.nice.org.uk); NCI: National Cancer Institute (http://www.cancer.gov/); BDI: Beck Depression Inventory; CI: Confidence Interval; GP: General practitioner; HTA: NHS National Institute for Health Research Health Technology Assessment programme (http://www.hta.ac.uk)

## Competing interests

JR and LD at the time of writing this paper were both employed by the NIHR Evaluation, Trials and Studies coordinating centre (http://www.netscc.ac.uk), who manage the NIHR HTA programme.

## Authors' contributions

LD conceived the analyses, checked the data extracted, classified the data, conducted the statistical analysis and wrote the first draft of the paper. JR raised the initial question about the proportion of trials with statistically significant results and carried out the preliminary analysis and provided advice throughout the study. All authors critically reviewed and agreed the final draft.

## Supplementary Material

Additional file 1 **Breakdown of the number of comparisons supplied by each trial**. A table showing why there are 85 primary comparisons from the 51 HTA superiority trials Click here for file

Additional file 2 **Characteristics of the 85 comparisons from the 51 included trials**. A table showing characteristics of the 85 primary comparisons included Click here for file
