# Supplementary material for: Treatment success in pragmatic randomised controlled trials: a review of trials funded by the UK Health Technology Assessment programme
Source: Trials. 2011 May 4;12:109. doi: 10.1186/1745-6215-12-109 (PMC3113983; doi:10.1186/1745-6215-12-109)
Supplement: Additional file 1 — Breakdown of the number of comparisons supplied by each trial. A table showing why there are 85 primary comparisons from the 51 HTA superiority trials [file 1745-6215-12-109-S1.PDF]

Additional file 1, appendix 1 - Breakdown of the number of comparisons supplied by each trial

**No. of comparisons**	**No. of trials**	**No. of comparisons from these trials**	**Reasons trials supplied more than one comparison**
1	39	39	N/A
2	6	12	Three 3 arm trials (2 new, 1 control) with 1 PO<br>One 2x2 factorial trial with 1 PO<br>One 4 arm trial (2 new and 2 control) with 1 PO. The primary comparisons are the two new treatments being compared to 1 of the controls<br>One 2 arm trial with 2 POs
4	4	16	One 5 arm trial (4 new, 1 control) with 1 PO<br>Two 3 arm trials (2 new, 1 control) with 2 PO<br>One 2 arm trial with 4 PO
6	1	6	One 3 arm trial with 2 domains of SF36 as primary outcome
12	1	12	One 3 arm trial (two new, one control) with 6 domains of SF-36 as PO
Grand Total	51	85	

Footnote: PO=Primary outcome
