# Supplementary material for: Treatment success in pragmatic randomised controlled trials: a review of trials funded by the UK Health Technology Assessment programme
Source: Trials. 2011 May 4;12:109. doi: 10.1186/1745-6215-12-109 (PMC3113983; doi:10.1186/1745-6215-12-109)
Supplement: Additional file 2 — Characteristics of the 85 comparisons from the 51 included trials. A table showing characteristics of the 85 primary comparisons included [file 1745-6215-12-109-S2.PDF]

Additional file 2, appendix 2 – Characteristics of the 85 comparisons from the 51 included trials

Characteristic	No. of Comparisons (%)
**Study design**	
Parallel	81 (95)
Factorial	4 (5)
**Number of arms**	
2 arms	41 (48)
3 arms	33 (39)
4 arms	4 (5)
5 arms	7 (8)
**Interventions**	
Service delivery	17 (2)
Education and training	13 (15)*
Psychological therapy	11 (13)
Surgery	9 (11)
Drug	8 (9)
Physical therapies	8 (9)
Social care	6 (7)
Diagnostic	5 (6)
Devices	4 (5)
Complementary therapies	2 (2)
Diet	1 (1)
Vaccines and biologicals	1 (1)
**Primary outcome**	
Quality of life measure	26 (31)*
Symptom score or measurement of depression/pain	21 (25)
Positive event rate (e.g. improvement in symptoms)	13 (15)
Adverse event rate (e.g. post operative nausea and vomiting)	11 (13)
Survival /mortality	7 (8)
Measurement of function	6 (7)
Other	1 (1)

Footnote: * This figure includes 12 comparisons from one trial which had SF-36 as the primary outcome
